# Supplementary material for: Refining sequence-to-activity models by increasing model resolution
Source: Bioinform Adv. 2026 Jul 31;6(1):vbag122. doi: 10.1093/bioadv/vbag122 (PMC13427759; doi:10.1093/bioadv/vbag122)
Supplement: vbag122_Supplementary_Data [file vbag122_supplementary_data.pdf]

Preprint Vol. , No. , pp. 1–15
doi: https://doi.org/10.1101/2025.01.24.634804
Advance Access Publication Date: 26 June 2025
Preprint

Supplementary information: Refining sequence-to-activity models by increasing model resolution

Nuria Alina Chandra,[1] Yan Hu,[2,3] Jason D. Buenrostro,[2,3]
Sara Mostafavi[1,4,*] and Alexander Sasse[1,5,*]

[1]Paul G. Allen School of Computer Science and Engineering, University of Washington, Seattle 98195, WA, USA, [2]Gene Regulation Observatory, Broad Institute of MIT and Harvard, Cambridge 02142, MA, USA, [3]Department of Stem Cell and Regenerative Biology, Harvard University, Cambridge, 02138, MA, USA, [4]Canadian Institute for Advanced Research, Toronto, MG51ZB, ON, Canada and [5]Center for Synthetic Genomics, Center for Molecular Biology Heidelberg (ZMBH), Heidelberg University, Heidelberg, 69120, Germany

[*]Corresponding authors: saramos@cs.washington.edu, a.sasse@zmbh.uni-heidelberg.de

© The Author 2025. Published by Oxford University Press. All rights reserved. For permissions, please e-mail: journals.permissions@oup.com

SUPPLEMENTARY METHODS

Composite loss functions

We compared regular and composite loss functions. Regular loss functions measured the error of the number of per base pair Tn5 prediction directly. These predictions were explicitly computed by multiplying the predicted profile probabilities with the predicted total counts (accessibility) from the two bpAI-TAC output heads, and then compared to the measured count data. As regular losses, we tested Poisson Negative Log Likelihood (PNLL) loss and mean squared error (MSE) . We also tested composite losses that contain a mixture parameter λ which represents the fractional weight of the profile loss compared to the total accessibility objective, consisting of MSE of logged counts:

$$(1-\lambda) \times MSE(\log(\hat{y}) - \log(y)) + \lambda \times Loss_{Profile}(\hat{e}, e)$$

When $\lambda = 1$ the model is trained only on the profiles, while when $\lambda = 0$ the model is solely trained on the total accessibility counts, i.e. AI-TAC. We assessed the predictive power of two composite losses: First, using the multinomial negative log likelihood (MNLL) for the profiles. Second, a composite loss of the MSE of logged counts and the cross entropy (CE) loss of the profiles.

The composite loss of the MSE of logged counts and the multinomial negative log likelihood (MNLL) of the profiles approximates the PNLL loss Avsec et al. 2021 [1]:

$$\begin{aligned} -\log(Poisson(k^{obs}, k^{pred})) \approx -\lambda \times \log(p_{mult}(k^{obs}|p^{pred}, n^{obs})) \\ + (\log(n^{obs}) - \log(n^{pred}))^2 \end{aligned}$$

with

$$p_{mult}(k^{obs}|p^{pred}, n^{obs}) = \frac{n^{obs}!}{k_1! \cdots k_d!} p_1^{k_1} \cdots p_d^{k_d}$$

We also evaluated the composite loss of the MSE of logged counts and the cross entropy (CE). We did this because if we use the above relationship and substitute in $p_m ult$, we can see that this composite loss represents a weighting of the cross entropy loss of each profile with the total counts of that region:

$$\begin{aligned} &\log(p_{mult}(k^{obs}|p^{pred}, n^{obs})) = \\ &\log(\frac{n^{obs}!}{k_1^{obs}!...k_d^{obs}!}) + \sum_{i=1}^{d} k_i^{obs} \cdot \log(p_i^{pred}) \\ &= \text{const.}^{obs} + \sum_{i=1}^{d} n^{obs} \frac{k_i^{obs}}{n^{obs}} \cdot \log(p_i^{pred}) \\ &= \text{const.}^{obs} + n^{obs} \sum_{i=1}^{d} p_i^{obs} \cdot \log(p_i^{pred}) \\ &= \text{const.}^{obs} + n^{obs} CRE(p_i^{obs}, p_i^{pred}) \end{aligned}$$

Seqlet extraction & clustering

We used the following criteria to determine sequences that were consistently well and better predicted in bpAI-TAC compared to AI-TAC in five model initializations:

- Strong signal: $max_{Celltype}$(accessibility) > 150.
- High variability across cell types: coefficient of variation > 1.
- Good prediction with bpAI-TAC: average Pearson correlation $R > 0.5$.
- Consistently better than AI-TAC: all ten initializations $R_{bpAI-TAC}$ > all trials ofR_{AI-TAC}

For the resulting 1,081 sequences in the test set, We used DeepSHAP [2] to compute the attributions for the 10 cell lineages by summing the predictions for the cell types in each lineage before applying DeepSHAP. We extracted motifs

from the attribution maps (i.e. seqlets), using rules about significance and number of subsequent base attributions. Hypothetical attribution scores (i.e. centered to zero at each position) for all four base-pairs were extracted for windows if it contained attributions for the present base that were above 1.96 of the standard deviation (equivalent to p-value < 0.05, two-tailed T-test) of attributions across all sequences, and the motif consisted of at least four of these significant bases allowing only for single insignificant base-pair gaps between significant positions. Seqlets were extracted from the attributions of bpAI-TAC and AI-TAC across all 10 lineages and then clustered with agglomerative clustering with complete linkage, using p-values of the strongest correlation of aligned motifs as a distance metric. Using p-values instead of correlations accounts for the length of the motif alignment. Clusters were assigned to motifs that shared at least a 0.05 p-value for their correlation with each other (Figure 3c).

SUPPLEMENTARY FIGURES

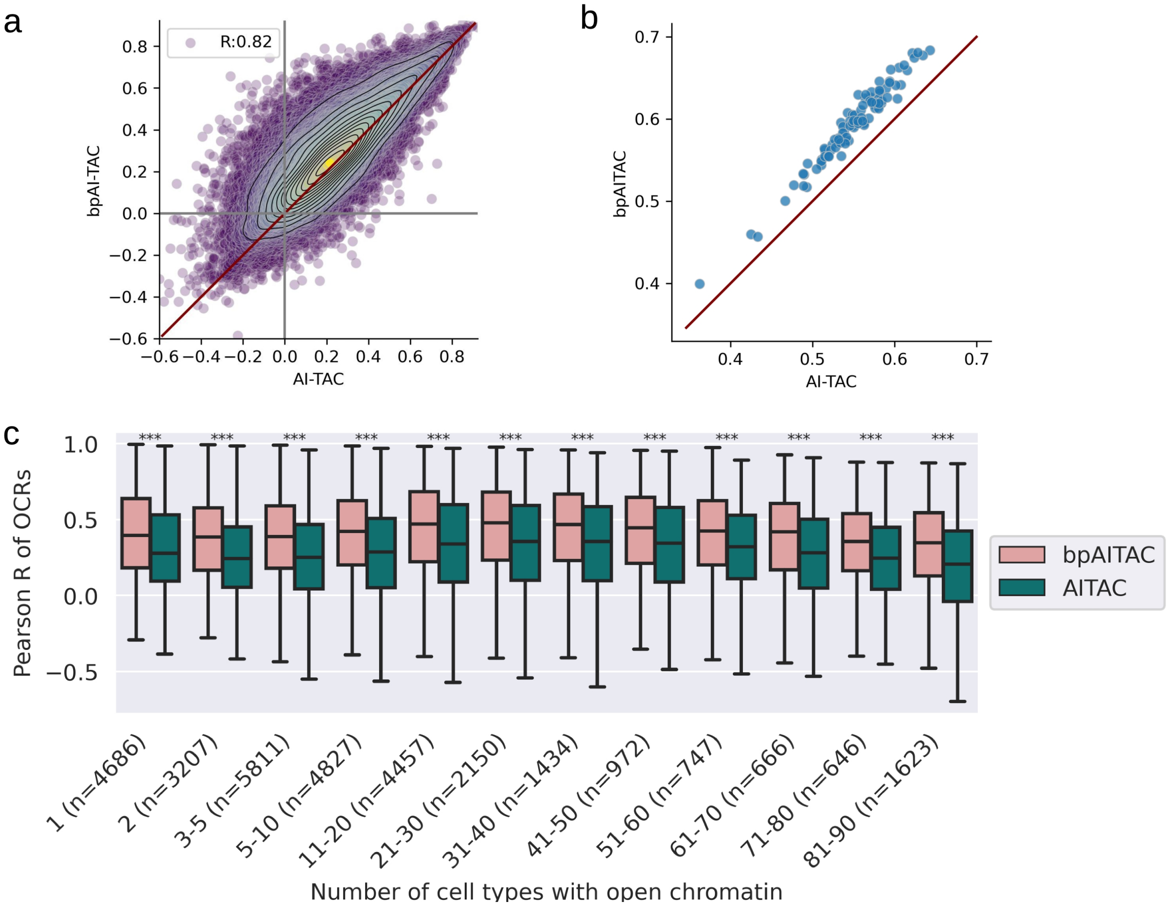

Fig. S1. a) Comparison of Spearman correlation of OCRS across cell types between bpAI-TAC and AI-TAC for predicted chromatin accessibility predictions on held-out OCRs. N=32,361, $p < e^{-100}$, Wilcoxon Signed-Rank Sum Test. b) Spearman correlation of cell types across held-out OCRs for bpAI-TAC and AI-TAC chromatin accessibility predictions. N=90 cell types, $p = 1.744e^{-16}$, Wilcoxon Signed-Rank Sum Test. c) Pearson correlation of OCRs across cell types for bpAI-TAC and AI-TAC accessibilty predictions binned by the number of cell types in which they are accessible (peak calling, $p < 0.05$).

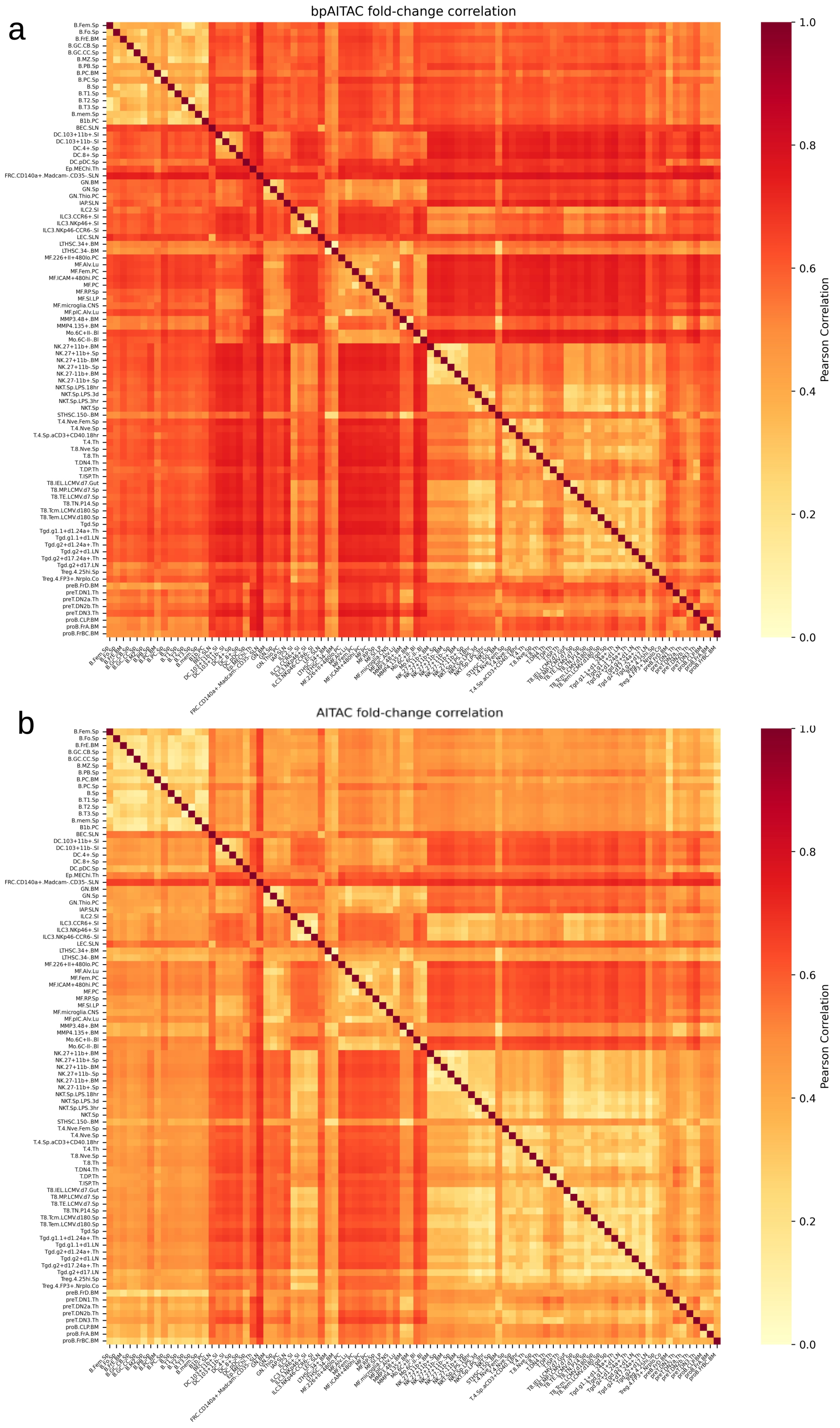

Fig. S2. Correlation matrices for predicted versus measured log2 changes between 4005 pairs of cell types. Only OCRs that are accessible in at least one of the two cell types (peak calling, $p < 0.05$) were included to compute correlations.

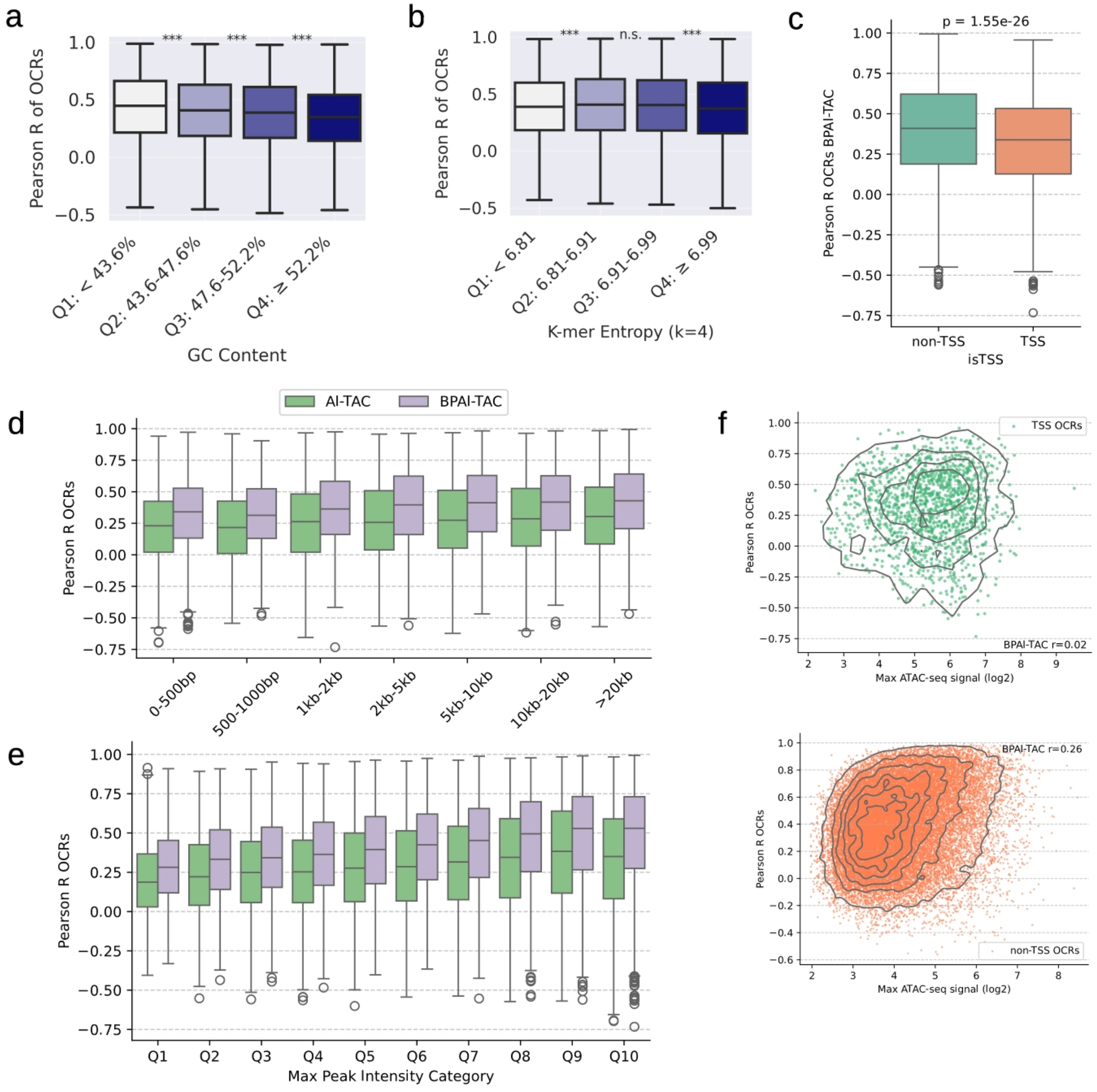

Fig. S3. Performance of bpAI-TAC stratified on sequence, location, and signal. a) Pearson correlation between predicted and measured accessibility of OCRs across cell types stratified on GC content. b) Pearson correlation between predicted and measured accessibility of OCRs stratified on 4-mer entropy (repetitiveness). c) Pearson correlation between predicted and measured accessibility of OCRs stratified on whether the OCR is located in proximity of a TSS (n=1,549) and non-TSS (n=30,812) (Mann-Whitney-U-Test). d) Comparison of Pearson correlation between predicted and measured accessibility of OCRs between bpAI-TAC and AI-TAC for categories based on the distance of the peak to the closest TSS. e) Comparison of Pearson correlation between predicted and measured accessibility of OCRs between bpAI-TAC and AI-TAC for OCRs deciles based on the maximum signal across cell types. f) Scatterplot for TSS (green) and non-TSS (coral) OCRs between the maximum signal across cell types and the Pearson correlation between predicted and measured accessibility.

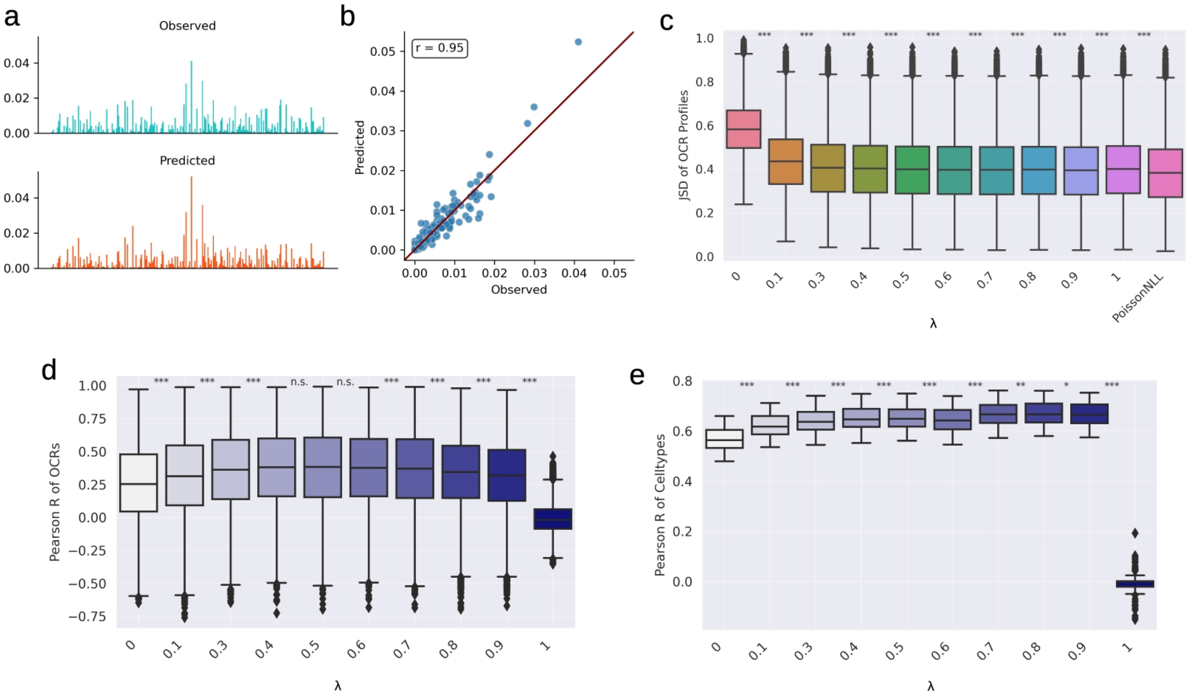

Fig. S4. Optimization of the accessibility-profile ratio lambda of the composite loss function (MSE+CE) a) Predicted Observed profile in single OCR chr11, 90,249,323 +-125, mm10 in cell type GN.Thio.PC, b) Scatter plot between the predicted per base probabilities in a). c) Jensen-Shannon divergence (JSD) distribution between predicted and observed Tn5 insertion profiles from models trained with composite loss functions combining mean squared error (MSE) and cross entropy (CE) loss. The contribution of profile loss was modulated by the profile loss fraction (λ). As a reference, all composite losses were compared to a model trained with Poisson Negative Log-Likelihood (PNLL) loss applied to per-base insertion counts reconstructed by multiplying the predicted total accessibility with the predicted profile probabilities. Wilcoxon Signed-Rank Sum Test. d) Pearson R of OCRs for different lambda ratios. Wilcoxon Signed-Rank Sum Test. e) Pearson R of Cell types for different lambda ratios. Wilcoxon Signed-Rank Sum Test.

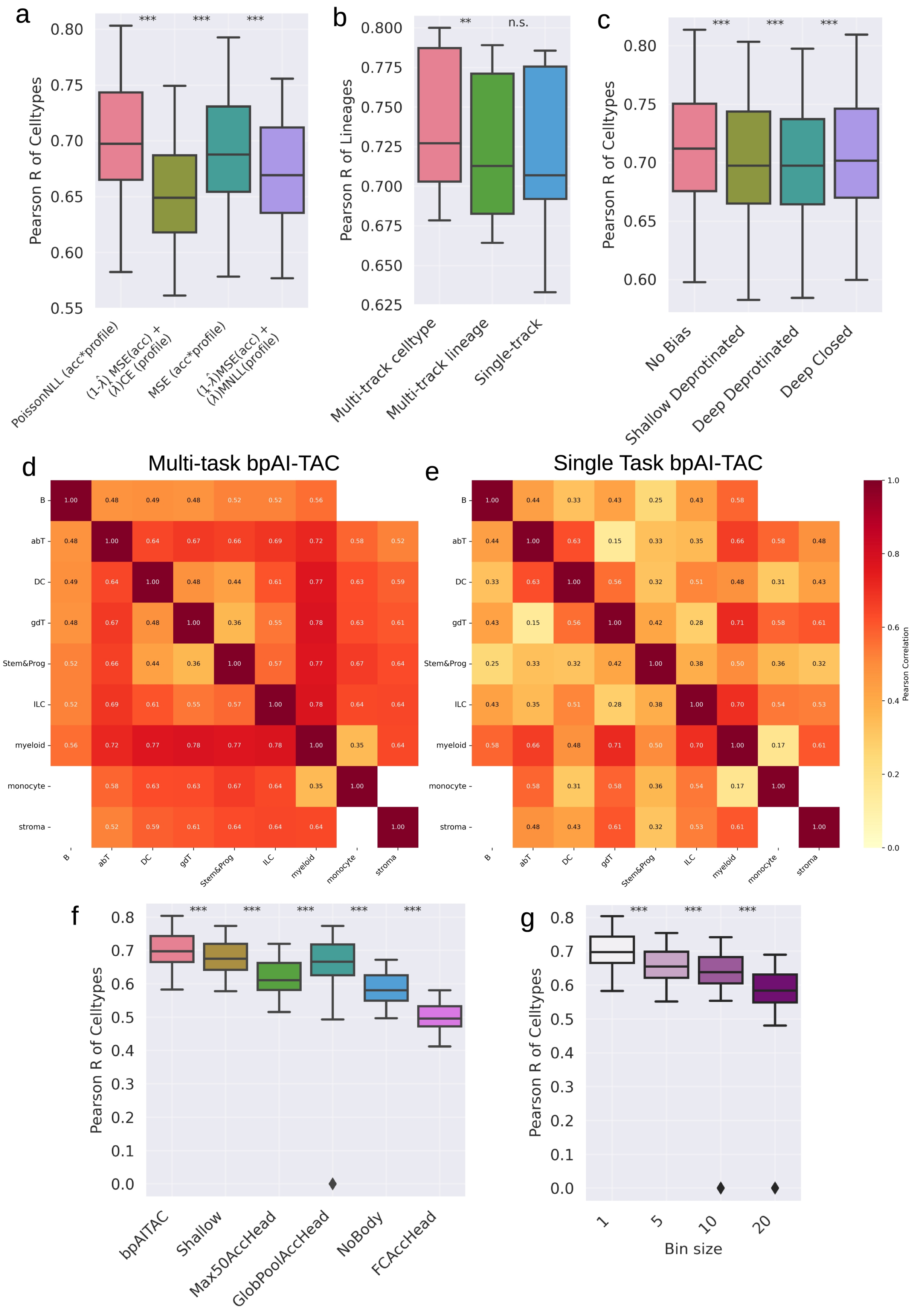

Fig. S5. Predictions across test set OCRs for different cell types.

Fig. S5. (cont.) a) Pearson R of cell types across OCRs for four loss functions. N=90, Signed-Rank-Test. b) Pearson R of cell lineages across OCRs. The Multi-Track Celltype model is trained to predict accessibility in 90 different cell types, with predictions averaged into 10 different lineages. The Multi-Track Lineage model is trained to predict accessibility across 10 different lineages. The Single-Track model is an ensemble of the results from 10 individual models trained on individual lineages. c) Pearson R of cell types across OCRs for bpAI-TAC trained using four different Tn5 bias prediction approaches. From left to right these approaches are 1) no Tn5 bias prediction added, 2) a shallow CNN trained on protein-free DNA, 3) a deep CNN trained on protein-free DNA, and 4) a deep CNN trained on closed OCR regions. N=90, Signed-Rank-Test. d) Correlation matrix for predicted versus measured log2-fold changes of accessibility between 45 pairs of cell lineages from the multi-task model. Only OCRs that are accessible in at least one of the two cell lineages (peak calling, $p < 0.05$, accessible in at least half of the cell types in the lineage) were included to compute correlations. e) Same as in d for the single cell lineage trained model. f) Pearson R of cell types for BpAI-TAC architecture ablations, including reducing the size of the body (Shallow and NoBody), and modifying the accessibility head (GlobPoolAccHead, Max50AccHead, FCAccHead). N=90, Signed-Rank-Test. g) Pearson R of cell types across OCRs for bpAI-TAC trained on different resolution profiles. Profiles were binned (summed over regions of sizes 5, 10, and 20) to create lower resolution Tn5 profiles. N=90, Signed-Rank-Test.

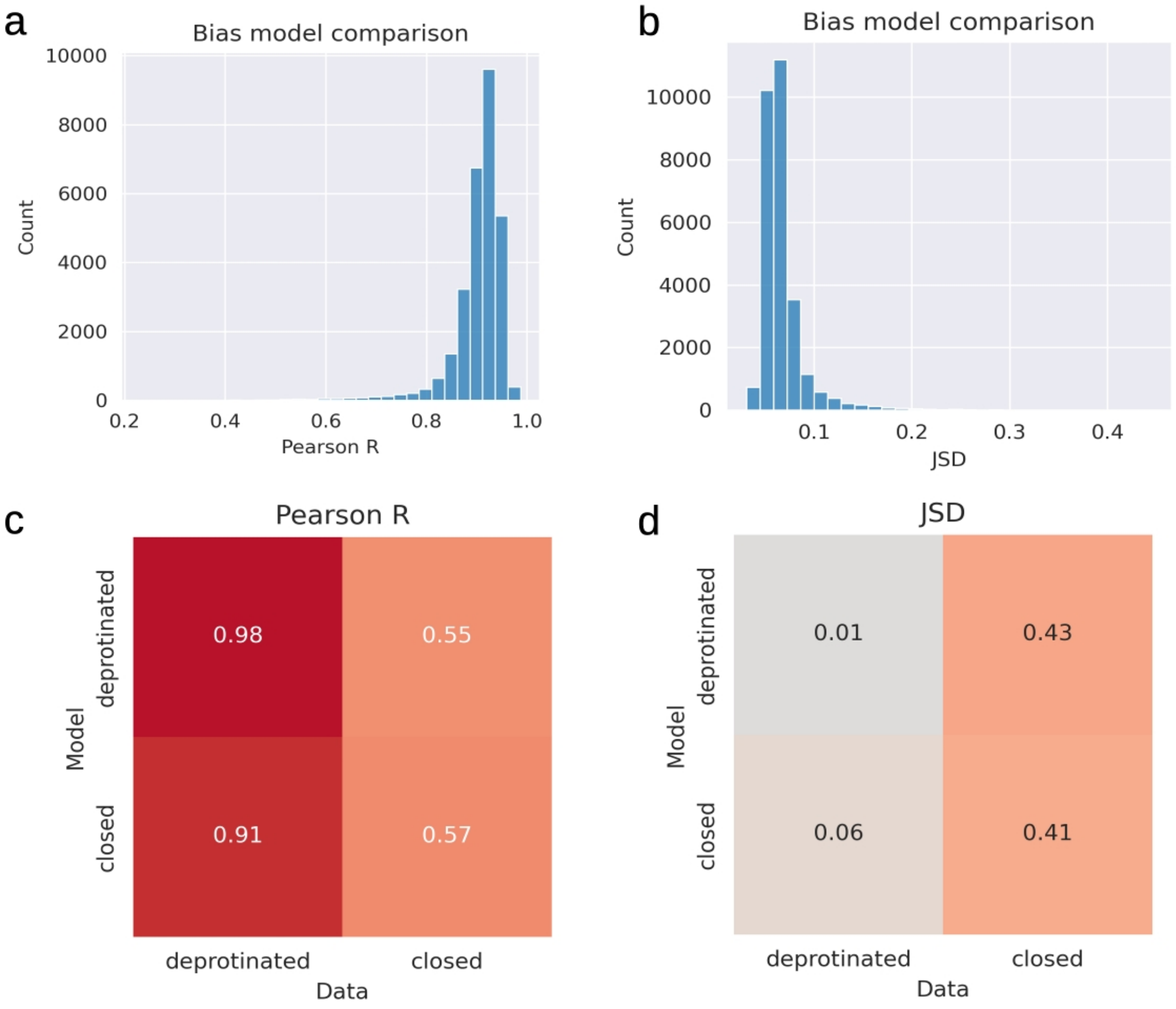

Fig. S6. Comparison of Tn5 bias prediction models trained on aggregated closed DNA regions and protein-free DNA. All analyses are performed on regions of DNA from held-out chromosomes. a) Mean Pearson R across profile predictions from two models trained on protein-free and aggregated closed regions on held-out test regions of closed regions of DNA. b) Jensen-Shannon divergence (JSD) between protein-free and closed bias model predictions on held-out test regions of closed regions of DNA. c) Pearson R of observed and predicted closed and protein-free DNA for both types of bias model. d) JSD of observed and predicted closed and protein-free DNA for both types of bias model.

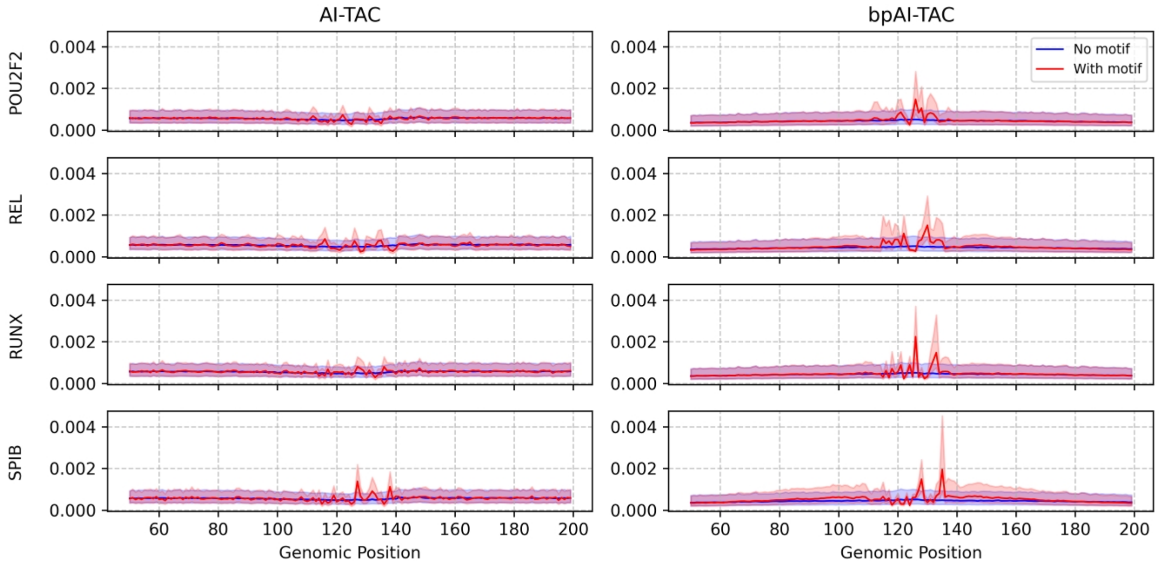

Fig. S7. AI-TAC and bpAI-TAC Tn5 bias subtracted profile predictions on 4000 dinucleotide shuffled sequences with motifs inserted (red), and controls without any motifs inserted (blue). The red and blue lines correspond to the median predicted values, and the translucent bands correspond to the interquartile range. Predictions are averaged across five model initializations.

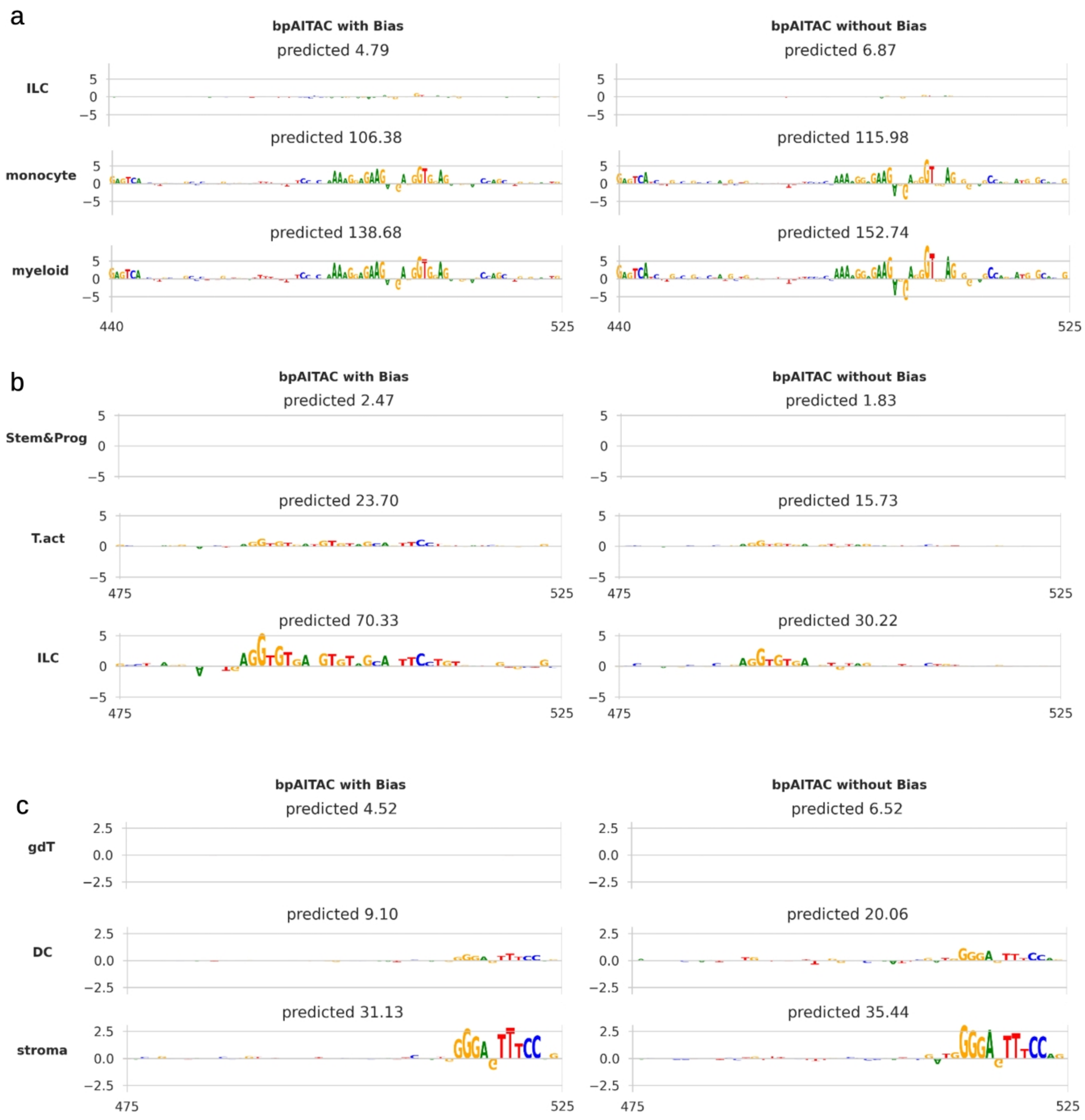

Fig. S8. Tn5 bias correction shows no major effect on bpAI-TAC's attribution maps for aggregated chromatin accessibility. Examples of bpAI-TACs attributions for chromatin accessibility of three regions, trained with and without the bias model. Attributions for the accessibility only show what contributes to the total number of Tn5 insertions, not the sequence elements that contribute to the shape of the profile.

Table S1: Per-celltype performance of bpAI-TAC. Pearson correlation, Spearman correlation, and Log MSE (base 2), across all regions in the test set $N = 32,361$. Pearson R of celltype specific open regions (peak calling, $p < 0.05$) in test set. Almost all celltypes have Pearson R in open regions above 0.55, with a mean of 0.622.

Cell Type	Pearson R	Spearman ρ	Log MSE	Pearson R of Open Regions [N]
B.Fem.Sp	0.707	0.595	2.547	0.581 [3392]
B.Fo.Sp	0.771	0.594	2.597	0.732 [6643]
B.FrE.BM	0.763	0.625	2.597	0.723 [6999]
B.GC.CB.Sp	0.700	0.603	2.810	0.633 [7702]
B.GC.CC.Sp	0.698	0.596	2.782	0.618 [6564]
B.MZ.Sp	0.709	0.562	2.726	0.629 [4552]
B.PB.Sp	0.752	0.546	2.355	0.685 [5250]
B.PC.BM	0.667	0.460	2.842	0.533 [2301]
B.PC.Sp	0.742	0.573	2.151	0.667 [5102]
B.Sp	0.747	0.567	2.776	0.693 [5291]
B.T1.Sp	0.752	0.608	2.402	0.708 [6800]
B.T2.Sp	0.765	0.586	2.517	0.710 [5489]
B.T3.Sp	0.773	0.596	2.522	0.724 [5890]
B.mem.Sp	0.766	0.606	2.337	0.728 [7799]
B1b.PC	0.704	0.546	2.956	0.621 [5507]
BEC.SLN	0.750	0.556	1.831	0.658 [4640]
DC.103+11b+.SI	0.784	0.639	2.018	0.745 [8157]
DC.103+11b-.SI	0.797	0.661	1.886	0.760 [8232]
DC.4+.Sp	0.773	0.634	1.858	0.738 [9052]
DC.8+.Sp	0.760	0.621	1.875	0.723 [8482]
DC.pDC.Sp	0.664	0.590	2.099	0.612 [7235]
Ep.MEChi.Th	0.773	0.633	1.632	0.721 [7726]
FRC.CD140a+.Madcam-.CD35-.SLN	0.693	0.617	1.773	0.572 [5471]
GN.BM	0.761	0.542	2.540	0.728 [5394]
GN.Sp	0.767	0.550	2.503	0.731 [5314]
GN.Thio.PC	0.746	0.597	2.382	0.711 [8321]
IAP.SLN	0.805	0.621	2.029	0.776 [8443]
ILC2.SI	0.628	0.522	3.253	0.498 [3945]
ILC3.CCR6+.SI	0.638	0.573	2.550	0.528 [5885]
ILC3.NKp46+.SI	0.641	0.522	2.831	0.536 [5109]
ILC3.NKp46-CCR6-.SI	0.636	0.533	2.639	0.522 [5229]
LEC.SLN	0.629	0.508	2.478	0.464 [2902]

Continued on next page

Table S1 – *Continued from previous page*

Cell Type	Pearson R	Spearman ρ	Log MSE	Pearson R of Open Regions [N]
LTHSC.34+.BM	0.665	0.553	2.608	0.578 [5206]
LTHSC.34-.BM	0.600	0.403	3.863	0.468 [2743]
MF.226+II+480lo.PC	0.725	0.682	1.880	0.683 [9775]
MF.Alv.Lu	0.719	0.612	2.134	0.660 [7410]
MF.Fem.PC	0.709	0.633	2.353	0.661 [8913]
MF.ICAM+480hi.PC	0.719	0.664	2.276	0.677 [9453]
MF.PC	0.731	0.650	2.196	0.689 [9325]
MF.RP.Sp	0.761	0.616	2.281	0.722 [7195]
MF.SI.LP	0.793	0.632	1.914	0.756 [8478]
MF.microglia.CNS	0.770	0.575	2.288	0.707 [4309]
MF.pIC.Alv.Lu	0.740	0.602	2.076	0.695 [8313]
MMP3.48+.BM	0.680	0.583	2.244	0.614 [5636]
MMP4.135+.BM	0.641	0.621	2.123	0.587 [8026]
Mo.6C+II-.Bl	0.714	0.645	1.738	0.646 [7707]
Mo.6C-II-.Bl	0.736	0.596	2.094	0.687 [7568]
NK.27+11b+.BM	0.689	0.599	2.225	0.604 [6407]
NK.27+11b+.Sp	0.680	0.579	2.283	0.602 [6678]
NK.27+11b-.BM	0.682	0.598	2.081	0.588 [6481]
NK.27+11b-.Sp	0.654	0.573	2.180	0.556 [6189]
NK.27-11b+.BM	0.698	0.567	2.352	0.606 [5139]
NK.27-11b+.Sp	0.695	0.536	2.315	0.610 [5512]
NKT.Sp.LPS.18hr	0.651	0.597	2.238	0.535 [4822]
NKT.Sp.LPS.3d	0.697	0.604	2.168	0.620 [6529]
NKT.Sp.LPS.3hr	0.658	0.556	2.358	0.553 [5615]
NKT.Sp	0.675	0.581	2.204	0.587 [5397]
STHSC.150-.BM	0.678	0.574	2.600	0.607 [5661]
T.4.Nve.Fem.Sp	0.701	0.606	2.119	0.609 [6180]
T.4.Nve.Sp	0.687	0.565	2.158	0.542 [3785]
T.4.Sp.aCD3+CD40.18hr	0.732	0.626	2.036	0.643 [6032]
T.4.Th	0.655	0.596	2.038	0.532 [5088]
T.8.Nve.Sp	0.666	0.616	1.927	0.558 [6595]
T.8.Th	0.657	0.622	2.015	0.535 [5791]
T.DN4.Th	0.728	0.681	1.734	0.646 [6744]
T.DP.Th	0.672	0.626	2.090	0.520 [3472]

Continued on next page

Table S1 – *Continued from previous page*

Cell Type	Pearson R	Spearman ρ	Log MSE	Pearson R of Open Regions [N]
T.ISP.Th	0.682	0.638	2.012	0.585 [5452]
T8.IEL.LCMV.d7.Gut	0.780	0.597	2.139	0.723 [5893]
T8.MP.LCMV.d7.Sp	0.727	0.626	2.113	0.639 [6495]
T8.TE.LCMV.d7.Sp	0.760	0.618	2.036	0.681 [6270]
T8.TN.P14.Sp	0.696	0.621	1.988	0.606 [6878]
T8.Tcm.LCMV.d180.Sp	0.626	0.572	2.515	0.520 [5983]
T8.Tem.LCMV.d180.Sp	0.640	0.543	2.538	0.528 [5038]
Tgd.Sp	0.682	0.620	2.051	0.581 [6085]
Tgd.g1.1+d1.24a+.Th	0.686	0.657	2.055	0.607 [6154]
Tgd.g1.1+d1.LN	0.630	0.570	2.500	0.491 [4129]
Tgd.g2+d1.24a+.Th	0.694	0.622	2.124	0.599 [4301]
Tgd.g2+d1.LN	0.643	0.552	2.455	0.530 [4600]
Tgd.g2+d17.24a+.Th	0.644	0.641	2.020	0.538 [5814]
Tgd.g2+d17.LN	0.611	0.458	3.304	0.448 [2188]
Treg.4.25hi.Sp	0.676	0.523	2.286	0.565 [4367]
Treg.4.FP3+.Nrplo.Co	0.739	0.574	2.348	0.656 [5405]
preB.FrD.BM	0.738	0.615	2.598	0.686 [6682]
preT.DN1.Th	0.621	0.665	1.930	0.557 [8730]
preT.DN2a.Th	0.582	0.630	2.108	0.512 [8515]
preT.DN2b.Th	0.604	0.641	2.024	0.523 [7774]
preT.DN3.Th	0.633	0.675	1.916	0.528 [6548]
proB.CLP.BM	0.694	0.676	1.754	0.653 [8904]
proB.FrA.BM	0.700	0.681	1.728	0.661 [9166]
proB.FrBC.BM	0.707	0.621	2.224	0.642 [6681]
**bpAI-TAC Mean**	**0.701**	**0.595**	**2.278**	**0.622**
**AI-TAC Mean**	**0.601**	**0.556**	**4.546**	**0.506**

REFERENCES

1. Žiga Avsec, Melanie Weilert, Avanti Shrikumar, Sabrina Krueger, Amr Alexandari, Khyati Dalal, Robin Fropf, Charles McAnany, Julien Gagneur, Anshul Kundaje, and Julia Zeitlinger. Base-resolution models of transcription-factor binding reveal soft motif syntax. *Nat. Genet.*, 53(3):354–366, March 2021.
2. Avanti Shrikumar, Peyton Greenside, and Anshul Kundaje. Learning important features through propagating activation differences. In Doina Precup and Yee Whye Teh, editors, *Proceedings of the 34th International Conference on Machine Learning*, volume 70 of *Proceedings of Machine Learning Research*, pages 3145–3153. PMLR, 2017.
